# Integration of transcriptome and proteome profiles in placenta accreta reveals trophoblast over-migration as the underlying pathogenesis

**DOI:** 10.1186/s12014-021-09336-8

**Published:** 2021-12-29

**Authors:** Na Li, Rui Hou, Caixia Liu, Tian Yang, Chong Qiao, Jun Wei

**Affiliations:** 1 Department of Obstetrics and Gynecology, Shengjing Hospital of China Medical University, Shenyang, China; 2 Key Laboratory of Maternal-Fetal Medicine of Liaoning Province; Key Laboratory of Obstetrics and Gynecology of Higher Education of Liaoning Province, Benxi, China; 3 Department of Obstetrics and Gynecology, West China Second University Hospital, Sichuan University, Chengdu, China

**Keywords:** Transcriptome, Proteome, Placenta accreta, Trophoblast, Migration

## Abstract

**Background:**

Placenta accreta (PA) is a major cause of maternal morbidity and mortality in modern obstetrics, few studies have explored the underlying molecular mechanisms.

**Methods:**

In our study, transcriptome and proteome profiling were performed in placental tissues from ten participants including five cases each in the PA and control groups to clarify the pathogenesis of PA.

**Results:**

We identified differential expression of 37,743 transcripts and 160 proteins between the PA and control groups with an overlap rate of 0.09%. The 33 most-significant transcripts and proteins were found and further screened and analyzed. Adhesion-related signature, chemotaxis related signatures and immune related signature were found in the PA group and played a certain role. Sum up two points, three significant indicators, methyl-CpG-binding domain protein 2 (MeCP2), podocin (PODN), and apolipoprotein D (ApoD), which participate in “negative regulation of cell migration”, were downregulated at the mRNA and protein levels in PA group. Furthermore, transwell migration and invasion assay of HTR-8/SVneo cell indicated the all of them impaired the migration and invasion of trophoblast.

**Conclusion:**

A poor correlation was observed between the transcriptome and proteome data and MeCP2, PODN, and ApoD decreased in transcriptome and proteome profiling, resulting in increased migration of trophoblasts in the PA group, which clarify the mechanism of PA and might be the biomarkers or therapy targets in the future.

**Supplementary Information:**

The online version contains supplementary material available at 10.1186/s12014-021-09336-8.

## Introduction

The incidence of placenta accreta has increased during the past few decades in China due to the two-child policy, leading to an increasing number of patients with a scarred uterus. Placenta accreta occurs when the placental chorionic villi adhere abnormally to or invade the uterine wall pathologically, and can be divided into three classes: placenta accreta, placenta increta, and placenta percreta [1, 2]. The prevalence of placenta accreta is reported to be 2–90 per 10,000 births [3], and the large range has been explained by different diagnostic criteria and different study populations. In our previous study, we reported that the incidence of placenta accreta in patients with a scarred uterus was 5.2% based on a clinical diagnosis [4]. Although placenta accreta is a major cause of maternal morbidity and mortality, few studies have explored the molecular mechanisms.

Proteomics technologies are key tools used to study complex biological processes at the protein level. Quantitative proteomics has been applied to modern obstetrics focusing on preeclampsia, recurrent miscarriage, gestational diabetes, and pre-term birth [5–9]. Different samples can be utilized for different purposes, including blood, plasma, placental tissues, amniotic fluid, and the umbilical artery to identify candidate biomarkers to predict the risk of a complicated pregnancy or to clarify the pathogenesis of a complicated pregnancy. Nevertheless, no reported attempts have been made to screen the differentially expressed proteins involved in the pathogenesis of placenta accreta.

Microarray-based transcriptional profiling is a powerful strategy for identifying disease-related genes and pathways [10], and a series of transcriptional analyses of placental tissues, decidual tissues, and fetal membranes from different gestational complications have been completed [11–15]. The placenta, as a transient organ that binds the mother and fetus, has a unique transcriptional landscape [16, 17]. Dysfunction of the placenta as a result of numerous changed genes is related to gestational complications. To the author’s knowledge, no study has performed a transcriptional profile of placenta accreta.

A hypothesis has been proposed that placenta accreta is associated with a decidual defect, trophoblast over-invasion, and abnormal angiogenesis [2]. Nevertheless, the results of the related studies focused on either transcripts or proteins, and there is a lack of correlation between RNA and proteins. Abdulghani performed a comparative analysis to uncover novel aspects of placental gene regulation during mouse placental development [18]. As the proteome and transcriptome reflect the expression of genes from two different levels, a combination of transcriptome and proteome profiling may provide new insight into biological processes related to disease development and to estimate transcriptional or post-transcriptional regulation, translational regulation, and protein translation efficiency in the pathogenic mechanism.

In this study, we analyzed placental tissues from five cases with placenta accreta and five control cases using RNA microarray and non-labeled quantitative proteomics technologies to clarify the pathogenesis of placenta accreta.

## Materials and methods

### Patients and sample collection

Placental tissues from placenta accreta and control pregnancies were collected immediately after cesarean deliveries at Shengjing Hospital, China Medical University, Shenyang, Liaoning. Maternal placental specimens containing villous and extravillous trophoblasts, the fibrinoid layer, and the basal plate layer were collected from placenta accreta (where placental tissues were tightly connected to the uterus and also included basal plate myometrial fibres) and control groups. The samples were washed in normal saline to remove excess blood, immediately immersed in liquid nitrogen, and transferred − 80 °C for storage.

Women were recruited if they met the following criteria: diagnosed with placenta previa complication based on ultrasonography, history of at least one previous caesarean section and pregnancy terminated by caesarean section.

The exclusion criteria were: multiple pregnancy, fetal anomalies, preterm premature rupture of the membranes, or infection and complications associated with any other obstetric disease, such as thyroid dysfunction, hypertension, and gestational diabetes. All participants enrolled in this study provided written informed consent.

The participants were divided into the placenta accreta group or the control group according to a placental pathology examination after the caesarean section. Cases in the placenta accreta group were diagnosed by pathologists (as shown in Additional file 1: Fig. S1).

Informed consent was obtained from patients and this study was approved by the ethical committee of Shengjing Hospital, China Medical University (No. 2017PS317K and 2017PS318K), according to the tenets of the Declaration of Helsinki.

### Cell lines, cell culture, and transfection

The immortalized human trophoblast cell line HTR-8/SVneo was cultured in RPMI 1640 medium (cat. no. 01-100-1A; Bioind, Beit Haemek, Israel) supplemented with 10% fetal bovine serum (FBS; cat. no. 04-001-1A; Bioind) (termed complete RPMI) at 37 °C in an atmosphere containing 5% carbon dioxide. For transient transfections, 1 × 10^6^ HTR-8/SVneo cells were plated into 6-well plates in complete RPMI. When the cells reached 60–70% confluency, si-MeCP2, si-PODN, and si-ApoD, or their negative controls (GenePharma, Suzhou, China) were transfected into the cells using Lipofectamine 3000 reagent according to the manufacturer's instructions (cat. no. L3000015; Invitrogen, Carlsbad, CA, USA). Cells were harvested for subsequent assays at 48 h after transfection.

### Extraction and analysis of RNA and proteins

Placental tissues (100 mg) were ground into a powder with a tissue grinder and lysed in Lysis Buffer (1 mM PMSF, 2 mM EDTA, and 10 mM DTT). Subsequently, the mixture was centrifuged at 25,000×*g* for 20 min at 4 °C, and the supernatant was added to 10 mM DTT in a water bath for 1 h at 56 °C. After returning to room temperature, 55 mM IAM was added in a dark room for 45 min followed by adding four volumes of cold acetone for 2 h at − 20 °C. The crude extract was incubated in Lysis Buffer (1 mM PMSF, 2 mM EDTA, and 10 mM DTT) again and cleared by centrifugation at 25,000×*g* for 20 min at 4 °C. The supernatant was collected and the protein concentration was measured by the Bradford quantitative assay. The extracted proteins were split to peptides, and peptides were equally mixed and diluted by buffer A (5% ACN, pH 9.8). Then, peptides were separated by liquid phase on LC-20AB liquid system (Shimadzu, Japan) with Gemini C18 column (4.6 × 250 mm, 5 μm). The buffers were applied using a gradient of 5% buffer B (95% CAN, pH 9.8) for 10 min, 5–35% buffer B for 40 min, 35–95% buffer B for 1 min, 50–100% buffer B for 5 min and lasted 3 min in buffer B followed by 5% buffer B for 10 min at a flow rate of 1 ml/min. Subsequently, the peptides were freeze-dried, redissolved by buffer A (2% CAN, 0.1% FA) and were separated by UltiMate 3000 UHPLC (Thermo Fisher Scientific, San Jose, CA). Samples were loaded on a home-made C18 column (150 μm ID × 25 cm) and eluted with following gradient in 3 h at a flow rate of 1 nl/min: 5% buffer B (98% ACN, 0.1% FA) in 0–5 min, 5–35% buffer B in 5–160 min, 35–80% buffer B in 160–170 min, 80% buffer B in 170–175 min and 5% buffer B in 176–180 min. The fractionated peptides were ionized by nanoESI source under 1.6 kV voltage and then measured by using LC–MS/MS on the tandem mass spectrometer Q-Exactive HF (Thermo Fisher Scientific). For the full MS scan, peptides were measured by Orbitrap analyzer, the mass range was set to 350–1500 mass/charge (m/z) and the resolution was 120,000 at m/z 200. Dynamic exclusion was used with 30.0 s duration.

Total RNA from placental tissues and cultured cells was extracted using TRIzol solution according to the manufacturer’s instructions (Invitrogen, Carlsbad, CA, USA) with strict quality control at each step.

The protein and RNA extraction and process were performed by BGI-Tech, and samples were sequenced using data independent acquisition (a non-labeled quantitative proteomics, also known as SWATH) for the proteome (ProteomeXchange Consortium via the iProX (IPX0002144000) partner repository with the dataset identifier PXD018665) and the Illumina HiSeq was used for the transcriptome (GSE148952).

### Western blotting

Placental tissues from the control and placenta accreta groups and cultured cells were lysed in radioimmunoprecipitation (cat. no. P0013B; Beyotime, Shanghai, China) buffer with 1% phenylmethylsulfonyl fluoride (PMSF; cat. no. ST506; Beyotime) on ice for 20 min. Then, the mixture was centrifuged at 12,000 rpm for 20 min at 4 °C to collect the supernatant. Protein concentrations were measured using the Pierce® BCA Protein Assay Kit-Reducing Agent Compatible (cat. no. 23225; ThermoFisher Scientific, Waltham, MA, USA). Then, 60 μg protein was separated by 10% sodium dodecyl sulfate polyacrylamide gel electrophoresis (cat. no. P0012A; Beyotime) and transferred to a PVDF membrane (cat. no. IPVH00010; Millipore, Darmstadt, Germany). After blocking with 5% non-fat milk in TBST containing 0.1% Tween 20 for 2 h, the membranes were incubated with primary antibodies overnight at 4 °C. The following primary antibodies were used: MeCP2 (1:1000; cat. no. 10861-1-AP; Proteintech), PODN ((1:1000; cat. no. 15014-1-AP; Proteintech), and ApoD (1:1000; cat. no. 10520-1-AP; Proteintech). The following day, the membranes were washed with Tris-buffered saline containing 0.1% Tween 20, and the HRP-conjugated secondary antibody (1:4000; cat. no. SA00001-1 for anti-mouse and cat. no. SA00001-2 for anti-rabbit; Proteintech) was incubated with the membranes for 90 min at room temperature. The proteins were detected using Immobilon Western HRP Substrate (cat. no. WBKLS0500; Millipore). GAPDH (1:4000; cat. no. 60004-1-Ig; Proteintech) was used as an internal control. The buffers used to dilute the antibodies was TBST containing 0.1% Tween 20.

### Quantitative reverse transcriptase polymerase chain reaction

Reverse transcription was performed using the PrimeScript RT Reagent Kit with the gDNA Eraser (cat. no. RR047A; TaKaRa). Then, mRNAs were quantified using TB Green® Premix Ex TaqTM II (Tli RNaseH Plus; cat. no. RR820A; TaKaRa). GAPDH was used to normalize the mRNA, and the results were analyzed using the ΔΔCt method. Primer sequences for the qRT‐PCR analysis are presented in Additional file 2: Table S1.

### Transwell migration and invasion assay

For the migration assay, a 100-μl cell suspension at a density of 2 × 10^5^ cells/ml with serum-free medium was placed in the upper chamber of a Transwell system (cat. no. 3422; Corning) and the lower chamber was filled with 600 μl RPMI 1640 medium (20% FBS). After a 24-h incubation, the cells were fixed with 4% paraformaldehyde, and crystal violet (cat. no. C0121; Beyotime) was applied for 30 min at room temperature to stain the cells. Cell numbers were quantified under a microscope (magnification, ×200).

For invasion assays, the upper chamber of a Transwell system was pre-coated with Matrigel before the cells were plated. Matrigel (cat. no. 354234; Corning), melted overnight at 4 °C, was diluted to 1 mg/ml with serum-free medium on ice. Then, 100 μl diluted Matrigel was added into the upper chamber and incubated at 37 °C for 5 h. Serum-free medium was added to the Transwells and then the invasion assays were carried out in the same manner as in the migration tests.

### Bioinformatics analysis

Genes that were differentially expressed between the placenta accreta and control groups (based on the criteria of an absolute fold-change > 2 and an adjusted *P*-value < 0.001) were identified using the R package DEGseq [19] and differentially expressed proteins (based on the criteria of an absolute fold-change > 1.5 and a *P*-value < 0.05) were identified using the R package MSstats [20]. The Metascape website and ClueGo were used to evaluate the functional implications, including Gene Ontology (GO) and Kyoto Encyclopedia of Genes and Genomes (KEGG) pathway analyses [21, 22]. The principal components analysis (PCA) and heatmap were completed with R packages to depict the expression patterns across patients. Functional gene sets were obtained from (http://amigo.geneontology.org/amigo).

### Statistical analysis

Student’s *t-*test was used to assess differences in the distribution of continuous data or the Mann–Whitney *U*-test was used if the data were not normally distributed. Spearman’s correlation analyses were conducted to identify the correlation between the transcriptome and proteome. All statistical analyses were performed using SPSS 24.0 (SPSS Inc., Chicago, IL, USA), GraphPad Prism 7 (GraphPad Software Inc., La Jolla, CA, USA), and R (https://www.r-project.org) software. A *P-*value < 0.05 was considered significant for two-tailed tests.

## Results

### Transcriptome and proteome profiles

**Fig. 1:**
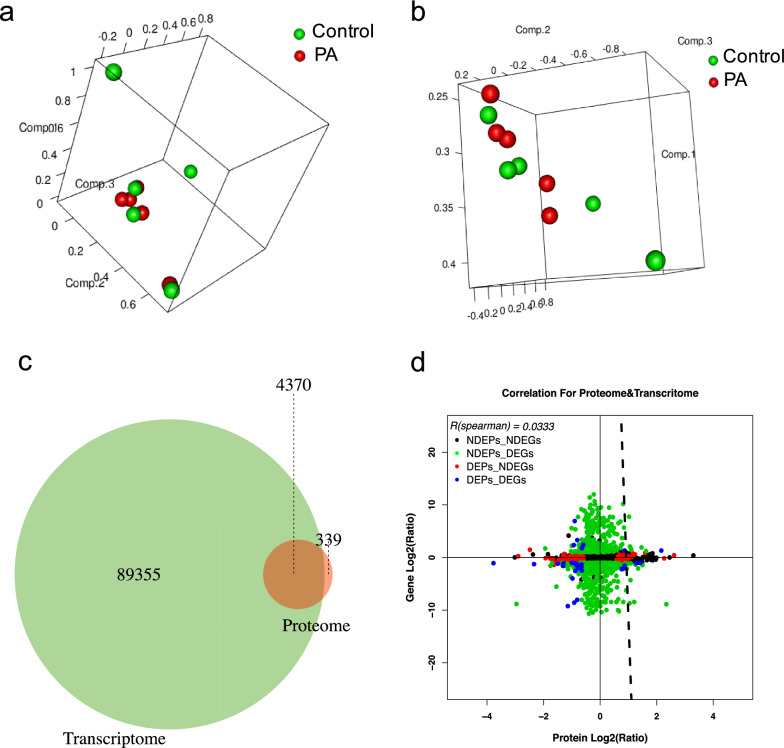
Basic profiling of the transcriptome and proteome. The PCA results showed the placenta accreta and control groups were generally distributed in different directions based on the transcriptome (**a**) and proteome (**b**), red dots denote PA group, green dots denote control group; **c** Venn diagram to reflect the 4.65% overlap degree of transcripts and proteins; **d** 2D plot of the correlation between the transcriptome and proteome which showed the correlation coefficient was 0.03, the x-axis is the protein expression level, and the y-axis is the gene expression level, black dots denote NDEPs_NDEGs, green dots denote NDEPs_DEGs, red dots denote DEPs_NDEGs, and bule dots denote DEPs_DEGs. PA: placenta accreta; NDEGs: not differentially expressed genes; NDEPs: not differentially expressed proteins; DEGs: differentially expressed genes; DEPs: differentially expressed proteins; PCA: principal components analysis

We identified 93,725 transcripts in the transcriptome and 4,709 proteins in the proteome of the placental tissues. The clinical information of the patients is summarized in Table 1. Moreover, the three-dimensional isomaps of the nonlinear PCA based on screened transcripts (Fig. 1a) and proteins (Fig. 1b) revealed that the placenta accreta and control groups were generally distributed in different directions. A Venn diagram was drawn to reflect the degree of overlap of the transcripts and proteins (Fig. 1c) and a correlation analysis was utilized to screen the genes from the 93725 transcripts and 4709 proteins between the transcriptome and proteome, resulting in a two-dimensional (2D) plot (Fig. 1d). There was a 4.65% overlap for all transcripts and proteins, and the correlation between the transcriptome and proteome was 0.03.

**Table 1:** Demographic and obstetrical characteristics

	PA n = 5	Control n = 5	*P* value
Age (years)	33.2 ± 6.83	33.4 ± 5.55	0.961
BMI	29.6 ± 1.85	28.0 ± 2.45	0.286
Gravity	3 (2–4)	3 (2–5)	0.841
Parity	1 (1)	1 (1–2)	0.690
Artificial abortion times	1 (0–2)	0 (0–1)	0.548
Gestational weeks	36.5 ± 0.57	36.5 ± 0.42	0.931

Values are presented as the mean ± standard deviation (SD) and median (range). PA: placenta accreta; BMI: body mass index

### The most-significant proteins in the placenta accreta group

**Fig. 2:**
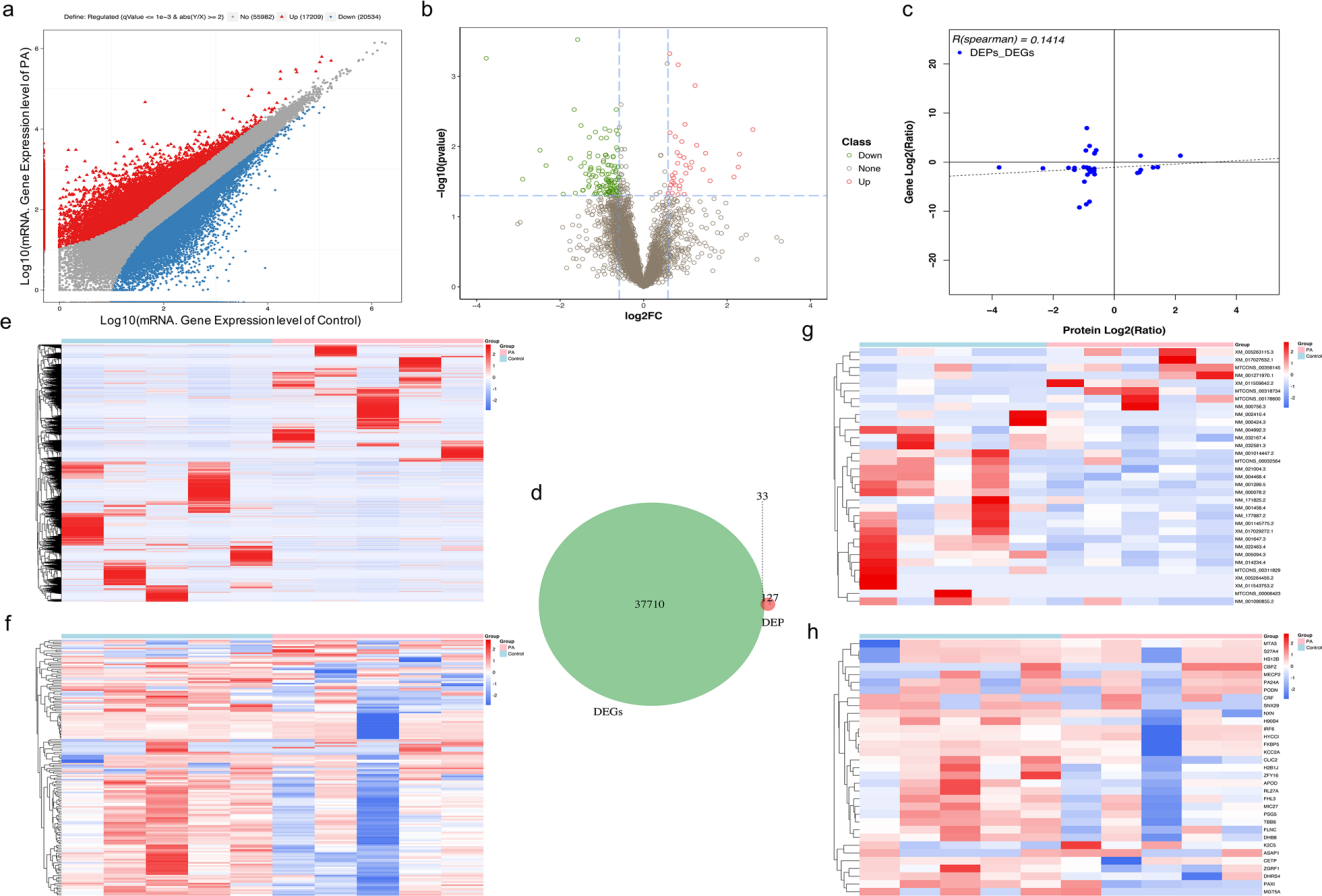
The most-significant proteins in the placenta accreta group. **a** Plot showing expression of transcripts, red triangles denote up-regulated genes, blue squares denote down-regulated genes, and grey dots denote non-regulated genes; **b** volcano plot of proteins, red dots denote up-regulated proteins, green dots denote down-regulated proteins, and grey dots denote non-regulated proteins, transverse dashed line denotes “−log10(0.05)”, vertical dashed line denotes “log2(0.67)” and “log2(1.5)” (from left to right); **c** 2D plot of the correlation between the DEGs and DEPs which showed the correlation coefficient was 0.14 (dashed line), the x-axis is the protein expression level, and the y-axis is the gene expression level; **d** Venn diagram to reflect the 0.09% overlap degree and 33 overlapping genes between the transcripts and proteins. Heatmaps of 37,743 transcripts (**e**) and 160 proteins (**f**), and heatmaps of 33 overlapping transcripts (**g**) and 33 proteins (**h**). FC: fold change; DEGs: differentially expressed genes; DEPs: differentially expressed proteins; PA: placenta accreta

To identify the significant genes or proteins in the pathogenesis of placenta accreta, the DEGseq R package was used, and the results revealed 37,743 differentially expressed transcripts (DEGs, 17,209 upregulated and 20,543 downregulated, Fig. 2a) and 160 differentially expressed proteins (DEPs, 38 upregulated and 122 downregulated, Fig. 2b) showed by volcano plot. However, a small overlap of 33 transcripts and 33 proteins was found between the DEGs and DEPs (Fig. 2d), and the 2D plot shows a 0.09% overlap in DEGs and DEPs (Fig. 2c). Similarly, the significant transcripts and proteins (Fig. 2e and f) and the 33 most-significant genes (Fig. 2g, h) were displayed well by the heatmaps between the placenta accreta and control groups. The 33 most-significant transcripts and proteins are shown in Table 2.

**Table 2:** The overlapped proteins and transcripts

Protein ID	DEPs	Gene ID	DEGs	Trend	SE
APOD	−	NM_001647.3	−	Same	0.35
ASAP1	+	MTCONS_00356145	+	Same	0.33
CBPZ	−	NM_001014447.2	−	Same	0.05
CETP	−	NM_000078.2	−	Same	0.28
CLIC2	−	NM_001289.5	−	Same	0.58
CRF	+	NM_000756.3	+	Same	0.70
DHB8	−	NM_014234.4	−	Same	0.42
DHRS4	−	NM_021004.3	−	Same	0.88
FHL3	−	NM_004468.4	−	Same	0.38
FKBP5	−	NM_001145775.2	−	Same	0.34
FLNC	−	NM_001458.4	−	Same	0.40
H2B1J	−	MTCONS_00311829	−	Same	0.43
H90B4	−	NM_001271970.1	+	Opposite	0.22
HS12B	−	XM_017027632.1	+	Opposite	0.42
HYCCI	+	NM_032581.3	−	Opposite	0.43
IRF6	+	MTCONS_00032564	−	Opposite	0.31
K2C5	+	NM_000424.3	−	Opposite	0.39
KCC2A	−	NM_171825.2	−	Same	0.25
MECP2	−	NM_004992.3	−	Same	0.72
MGT5A	+	NM_002410.4	−	Opposite	0.24
MIC27	−	XM_017029272.1	−	Same	0.38
MTA3	−	XM_005264456.2	−	Same	0.46
NXN	−	NM_022463.4	−	Same	0.42
PA24A	−	XM_011509642.2	+	Opposite	0.35
PAXI	−	NM_001080855.2	−	Same	0.34
PODN	−	MTCONS_00006423	−	Same	0.25
PSG5	−	MTCONS_00178600	+	Opposite	0.28
RL27A	−	MTCONS_00318734	+	Opposite	0.43
S27A4	−	NM_005094.3	−	Same	0.33
SNX29	+	NM_032167.4	−	Opposite	0.50
TBB8	−	NM_177987.2	−	Same	0.19
ZFY16	−	XM_011543753.2	−	Same	0.33
ZGRF1	−	XM_005263115.3	+	Opposite	0.29

DEG: differentially expressed gene; DEP: differentially expressed proteins; SE: sensitivity; “+”: up-regulated compared with the control group; “−”: down-regulated compared with the control group

### Enhanced adhesion signature in placenta accreta

**Fig. 3:**
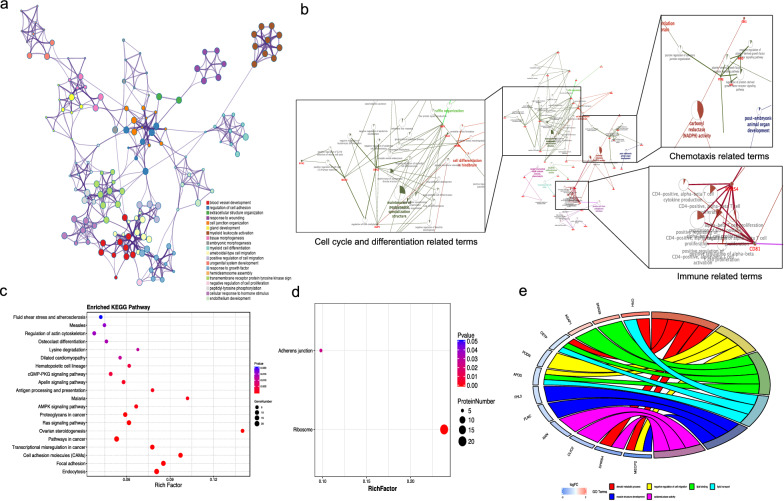
The placenta accreta-mediated enhanced adhesion-related terms. GO analysis based on top 500 transcripts showing the top 20 GO terms which revealed that blood vessel development and cell adhesion-related terms were most enriched (**a**) and significant proteins which showed the cell cycle and differentiation, chemotaxis and immune-related terms were enriched (**b**). KEGG analysis based on the top 500 transcripts showing the top 20 KEGG pathway (**c**) and significant proteins (**d**) which revealed that the focal adhesion-related pathway was enhanced in the placenta accreta group. GO analysis based on the 33 most-significant proteins showing increased cell migration pathway (**e**). FC: fold change

To clarify the biological features of the transcriptome and proteome, we performed GO and KEGG analyses based on the significant genes or proteins. We used the analyses among the top 500 significant transcripts according to the adjusted P-value (Additional file 2: Table S2). The results revealed that blood vessel development and cell adhesion-related terms were most enriched in the GO analysis (Fig. 3a). Furthermore, the cell cycle and differentiation, chemotaxis and immune-related terms were enriched in the significant proteins (Fig. 3b, significant proteins are shown in Additional file 2: Table S3). The KEGG results revealed that the focal adhesion-related pathway was enhanced based on the top 500 significant transcripts in the placenta accreta group, which was similar with the results in the proteome (Fig. 3c and d). In addition, the cell migration pathway increased in the most-significant proteins (Fig. 3e).

### Validation of proteins involved in the adhesion related pathway

**Fig. 4:**
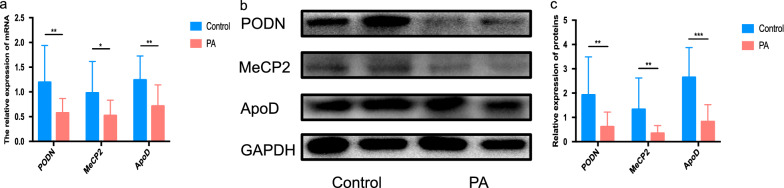
Analysis of MeCP2, PODN, and ApoD expression in placental tissues. **a** mRNA expression showed that MeCP2, PODN, and ApoD were downregulated in the placenta accreta group by qRT-PCR; **b**, **c** protein levels showed that MeCP2, PODN, and ApoD were downregulated in the placenta accreta group based on western blot and the statistical results. All data are presented as mean ± standard deviation. PA: placenta accreta; MeCP2: Methyl-CpG-binding domain protein 2; ApoD: Apolipoprotein D; PODN: Podocan. *P < 0.05, **P < 0.01, ***P < 0.001

Based on the GO-BP process (Fig. 3e) and critical role of adhesion-related signature, we analyzed all proteins in the “negative regulation of cell migration” and overlapped with the most-significant proteins, which revealed that MeCP2, PODN, and ApoD might be major proteins in the signature to promote the development of placenta accreta. Therefore, PCR was performed to estimate the mRNA (Fig. 4a) and protein levels (Fig. 4b and c) of this gene. The results showed that MeCP2, PODN, and ApoD were downregulated in the placenta accreta group (*P* = 0.02, *P* = 0.009, *P* = 0.004, respectively in mRNA level and *P* = 0.0012, *P* = 0.0046, *P* = 0.0003, respectively in protein level).

### MeCP2, PODN and ApoD downregulation promote migration and invasion of HTR-8/SVneo cells

**Fig. 5:**
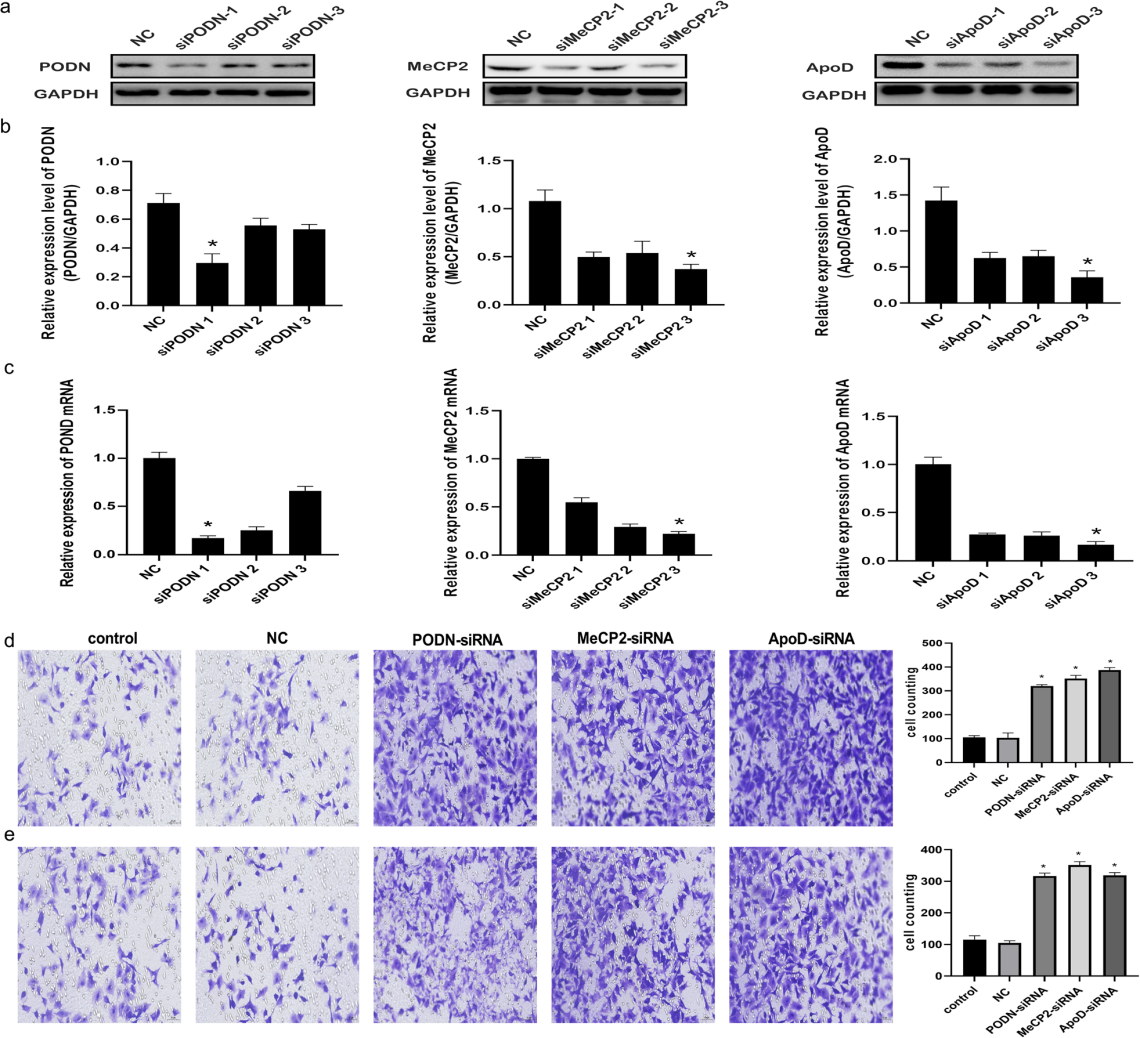
MeCP2, PODN and ApoD downregulation promotes the migration and invasion of HTR-8/SVneo cells. **a**, **b** Levels of MeCP2, PODN and ApoD proteins detected after transfection of HTR-8/SVneo cells with siRNA for MeCP2, PODN and ApoD, respectively. **c** MeCP2, PODN and ApoD mRNA expression examined after 48-h transfection. **d**, **e** Transwell migration and invasion assays showing higher numbers of migrated and invaded cells in the si-MeCP2, si-PODN and si-ApoD group than in the NC group (×200). All data are presented as the mean ± standard deviation. NC: negative control; MeCP2: Methyl-CpG-binding domain protein 2; ApoD: Apolipoprotein D; PODN: Podocan. *P < 0.05

To clarify the function of MeCP2, PODN and ApoD, si-MeCP2, si-PODN, and si-ApoD, or their negative controls were transfected into HTR-8/SVneo cell lines. After 48 h, si-MeCP2, si-PODN and si-ApoD resulted in decreased protein expression level of them (Fig. 5a and b). Additionally, the mRNA expression levels of MeCP2, PODN and ApoD confirmed that the transfection was effective (Fig. 5c) and the most effective siRNAs were selected for future research. Furthermore, Transwell experiments performed to explore the function of MeCP2, PODN and ApoD showed that si-MeCP2, si-PODN and si-ApoD significantly increased the migration and invasion rate of HTR-8/SVneo cells (Fig. 5d and e).

## Discussion

In our study, we screened significant transcripts and proteins with a limited 0.09% overlap between them in the placenta accreta group. Combining the transcriptome and proteome is more likely to detect a significant correlation when evaluating the mechanism and studying biomarkers [23–25]. It might be explained by the complex regulatory mechanism of the gene expression process. Analyzing the protein and mRNA results with the same significant differential expression trend can help confirm the sequencing results and explain the regulation of gene expression. The post-transcriptional regulatory mechanism and protein translation efficiency can be analyzed from the proteins and mRNAs with opposite expression changes. In other words, the complementary transcriptome and proteome are more beneficial to study phenotypic and genetic regulatory mechanisms in biological models [26]. Thus, we combined transcriptome and proteome profiling to clarify the mechanism regulating placenta accreta.

Although a poor correlation was observed between the transcriptome and proteome, there were still several common features: the adhesion-related signature increased in the transcriptome and proteome based on the KEGG and GO pathway analyses in the placenta accreta group, demonstrating that trophoblast invasion plays a critical role in the development of placenta accreta, as shown in Additional file 1: Fig. S1. The boundary between trophoblasts and myometrium was clear in control group, while in placenta accreta group, trophoblasts invaded into the myometrium, indicating the enhanced invasion ability of trophoblasts in placenta accreta group.

Placental development is a complex and strictly controlled process. During pregnancy, cytotrophoblasts proliferate and differentiate by two ways: syncytiotrophoblasts or extravillous trophoblasts (EVT). Extravillous trophoblasts differentiate through two pathways: interstitial EVT that invades the decidua and myometrium of the uterus and intravascular EVT that invades the spiral artery with the characteristics of endothelial cells. Shallow invasion increases the risk of pregnancy complications, such as severe preeclampsia and fetal growth restriction (FGR) and so on. Overly deep invasion will lead to the hallmark of the placenta accreta.

Yue Chen reported that trophoblast cell adhesion and migration increase in MARVELD1 knockout mice, which exhibit a placenta accreta phenotype [27]. Furthermore, extravillous trophoblasts from patients with placenta accreta show more mesenchymal characteristics and lose E-cadherin, which is a transmembrane protein involved in cell adhesion [28–30]. Kocarslan reported that matrix metalloproteinase 2 is highly expressed in placenta percreta compared to normal placental tissues [31]. All of these results are consistent with the results in this study; the adhesion-related signature was enhanced in placenta accreta. In addition to EVT, Leah McNally found that overexpression of DOCK4 increased CTB invasiveness [32]. Our study further demonstrates over-invasion of trophoblasts in the pathogenesis of placenta accreta. It has been reported that in vitro model, undifferentiated endometrial stromal cells (ESCs) can promote the invasion of trophoblasts [33]. Moreover, decidual stromal cells (DSCs), which accounts for 75% of the total decidual tissue, can regulate trophoblast invasion [34]. However, due to the complexity of decidual tissue, in addition to DSC, there are decidual natural killer (NK) cells (natural killer cells), macrophages and so on, which makes the regulatory mechanism of decidual tissue on the trophoblast invasion unclear.

Our study also found that the immune-related terms were enriched at the proteomic level in the placenta accreta group, indicating that the immune signature may be involved in the pathogenesis of placenta accreta. Schwede reported that Treg-cells might regulate trophoblast invasion [35]. The invasion of trophoblasts is regulated by numerous factors and may be regulated by decidual cells, T cells, and NK cells at the maternal–fetal interface in addition to self-secretion. Abnormal immune function in the placenta may cause dysregulated communication among T cells, NK cells, and lymphocytes and their adequate interaction with each other may be a mechanism to ensure extravillous trophoblast invasion. Abnormal placental immune function in patients with placenta accreta may lead to an imbalance between them, resulting in excessive trophoblast invasion and promoting the occurrence of placenta accreta.

To clearly illuminate the pathogenic mechanism of placenta accreta, the most-significant proteins were identified and a functional analysis was performed. In this study, 33 proteins were most important, and the functional analysis revealed that the “negative regulation of cell migration” was enriched, which was similar with the functional analysis of the transcriptome and proteome. According to the “negative regulation of cell migration” process, we examined three proteins, such as apolipoprotein D (ApoD), podocan (PODN), and methyl-CpG-binding domain protein 2 (MeCP2), to demonstrate the veracity of profiling and migration signature involvement in the development of placenta accreta. Furthermore, the cell experiments confirmed the functions of MeCP2, PODN, and ApoD in trophoblast migration and invasion.

PODN, a new member of the small leucine-rich repeat protein family, has been reported to suppress migration [36, 37]. In our study, decreased expression of PODN promoted migration of the trophoblast leading to the development of placenta accreta. Previous studies have reported that PODN regulates Wnt/β-catenin signaling, which plays a critical role in cellular migration and embryonic development [36, 38, 39]. We speculated that PODN exerts its function in the extracellular matrix through Wnt/β-catenin signaling to mediate differentiation and migration of the trophoblast.

MeCP2 is an epigenetic regulator that binds to methylated CpG dinucleotide in DNA and is essential for embryo viability and placental development [40]. Cao et al. reported that MeCP2 is downregulated leading to dysregulation of expression of canonical transient receptor potential 3 in placental tissues of patients complicated with gestational diabetes mellitus [41]. Previous studies have reported that DNA methylation is critical during early trophoblast development and differentiation [42, 43]. It has been hypothesized that DNA hypomethylation allows upregulation of epithelial to mesenchymal transition (EMT) genes, which mediates acquisition of an invasive and migratory phenotype [44, 45]. In our study, MeCP2 decreased in the placenta accreta group indicating a hypomethylated state in placental tissues, which could influence the expression of genes involved in regulating cell proliferation, cell apoptosis, and cell invasion [45–47]. Downregulated MeCP2 in a patient with placenta accreta might affect the EMT or induce adhesion-related proteins to promote trophoblast invasion.

ApoD is a member of the lipocalin superfamily with a wide distribution in mammalian tissues and an apparent multifunctional role regulating lipid transport and lipid binding. Chih-Jen Lai reported that ApoD colocalizes with the high density lipoprotein (HDL) receptor (scavenger receptor, class B, type I, and SR-B1) to suppress formation and migration of human umbilical vein endothelial cells [48]. Additionally, very low density lipoprotein (VLDL) and LDL promote cell proliferation, metastasis, and angiogenesis and HDL glycation activates cell proliferation and migration and inhibits apoptosis in breast cancer [49, 50]. ApoD mediates binding of HDL to LDL in T24 carcinoma [51], and ApoD may regulate trophoblast migration by regulating LDL and HDL.

In summary, we explored the mechanism of placenta accreta and identified proteins that promote migration of the trophoblast. This is the first study to combine the transcriptome and proteome to elucidate the pathogenesis of placenta accreta and show that adhesion-related signatures increased in placenta accreta patients. However, several limitations of this study should be discussed. Only ten samples were assessed and only a single sample per patient was used in the third-trimester after delivery. Nevertheless, the small number of samples may have resulted from the low incidence of placenta accreta among pregnancies and the strict diagnostic criteria based on the pathological examination. Future multi-omics studies must be performed on a large cohort of patients and on spatially and developmentally distinct placental tissues in each patient to consider the inter- and intra-heterogeneity of placenta accreta. Furthermore, future research that include the analysis of maternal plasma collected during the pregnancy should be performed to identify the biomarkers based on the proteins that play a critical role in the pathogenesis of placenta accreta.

## Conclusion

A poor correlation was observed between the transcriptome and proteome data and MeCP2, PODN, and ApoD decreased in transcriptome and proteome profiling, resulting in increased migration of trophoblasts in the placenta accreta group, which clarify the mechanism of PA and might be the biomarkers or therapy targets in the future.

## Supplementary Information

**Additional file 1: Figure S1.** HE staining images of placental tissues. a and b, the HE staining images in control group, a indicates × 40 and b indicates × 200. c and d, the HE staining images in placenta accreta group, c indicates × 40 and d indicates × 200.

**Additional file 2.** The additional tables.

## Data Availability

The datasets during and/or analysed during the current study available from the corresponding author on reasonable request.
