# Supplementary figures and images for: Integration of transcriptome and proteome profiles in placenta accreta reveals trophoblast over-migration as the underlying pathogenesis
Source: Clin Proteomics. 2021 Dec 29;18:31. doi: 10.1186/s12014-021-09336-8 (PMC8903580; doi:10.1186/s12014-021-09336-8)

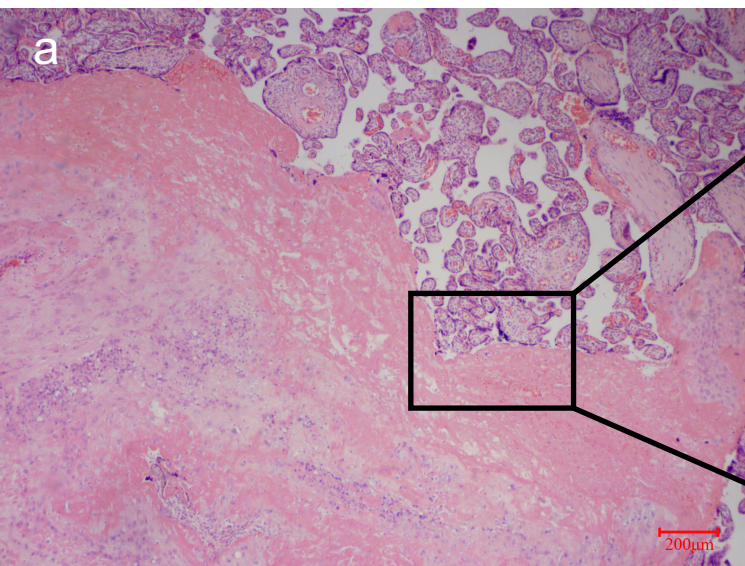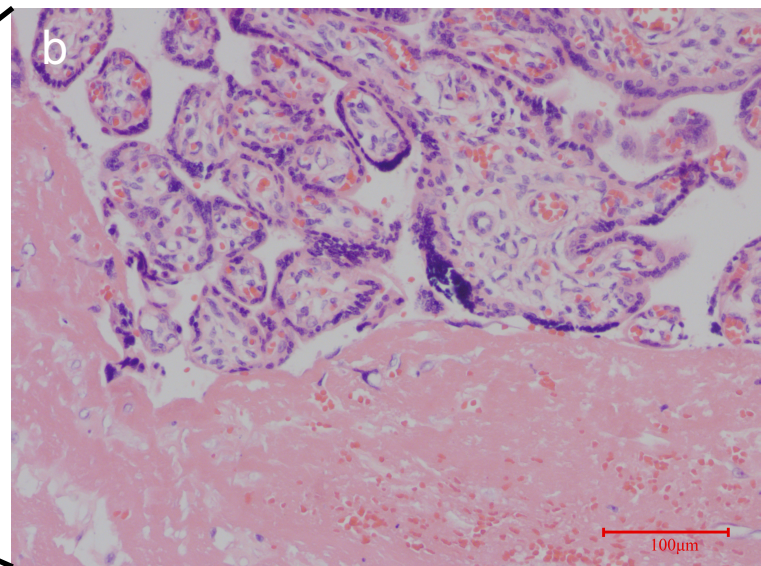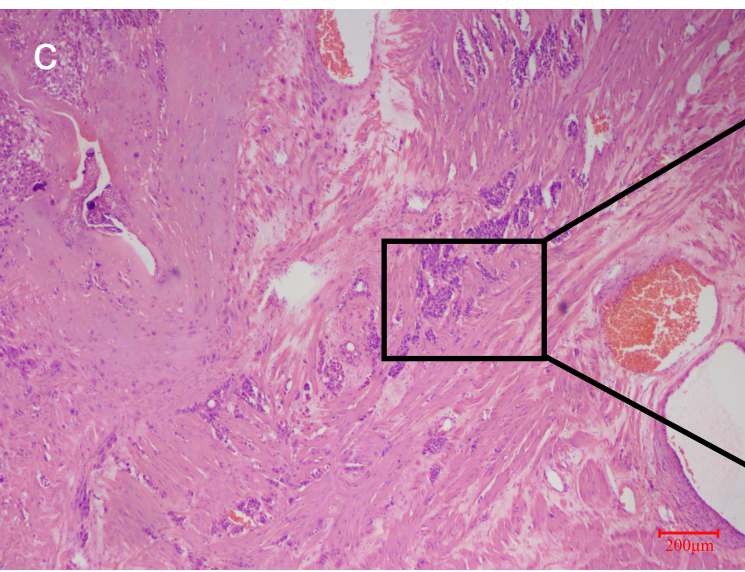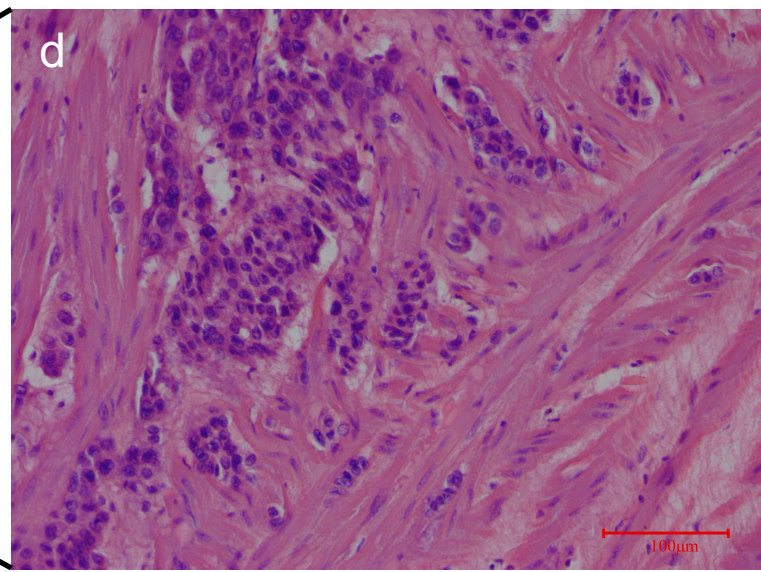

Supplement: Supplementary file 1 — Additional file 1: Figure S1. HE staining images of placental tissues. a and b, the HE staining images in control group, a indicates × 40 and b indicates × 200. c and d, the HE staining images in placenta accreta group, c indicates × 40 and d indicates × 200. [file 12014_2021_9336_MOESM1_ESM.pdf]
