# Supplementary material for: Integration of transcriptome and proteome profiles in placenta accreta reveals trophoblast over-migration as the underlying pathogenesis
Source: Clin Proteomics. 2021 Dec 29;18:31. doi: 10.1186/s12014-021-09336-8 (PMC8903580; doi:10.1186/s12014-021-09336-8)
Supplement: Supplementary file 2 — Additional file 2. The additional tables. [file 12014_2021_9336_MOESM2_ESM.docx]

Table S1. Primer sequences for qRT‐PCR.

| Gene | Forward Premier | Reverse Premier |
| --- | --- | --- |
| ApoD | GCTGGAAGTTAAGTTTTCCTGG | GGATGATGCAGGTACAGGAATA |
| PODN | TCCGTGGTGGACAGTGCCTTC | TCCTCCTCCTCTTCCTCCTCCTC |
| MeCP2 | ACCACCATCACCACCACTCAGAG | GACGCTGCTGCTCAAGTCCTG |
| GAPDH | ACAACTTTGGTATCGTGGAAGG | GCCATCACGCCACAGTTTC |

Table S2: The top 500 significant transcripts between placenta accreta and control group in transcriptome.

| Gene ID | log_2_FC | Regulation Trend | *P* value |
| --- | --- | --- | --- |
| NM_000517.4 | 2.40 | Up | 0 |
| XM_005246408.1 | 3.82 | Up | 0 |
| NM_000558.4 | 3.01 | Up | 0 |
| NM_000518.4 | 2.73 | Up | 0 |
| XM_005246404.1 | 3.22 | Up | 0 |
| XM_005246399.1 | 4.17 | Up | 0 |
| NM_002256.3 | 1.58 | Up | 0 |
| NM_006732.2 | 3.84 | Up | 0 |
| NM_003836.6 | -1.92 | Down | 0 |
| NM_000599.3 | -2.07 | Down | 0 |
| NM_000559.2 | 9.95 | Up | 0 |
| XM_005246409.1 | 4.28 | Up | 0 |
| XM_017002023.1 | -2.16 | Down | 0 |
| NM_000014.4 | -1.23 | Down | 0 |
| XM_006717129.2 | -1.16 | Down | 0 |
| NM_015103.2 | -5.62 | Down | 0 |
| NM_002293.3 | -1.40 | Down | 0 |
| NM_000435.2 | -1.14 | Down | 0 |
| NM_005346.4 | 1.24 | Up | 0 |
| XM_017024738.1 | -1.23 | Down | 0 |
| NM_001795.4 | -1.15 | Down | 0 |
| NM_012072.3 | -1.14 | Down | 0 |
| NM_000442.4 | -2.73 | Down | 0 |
| NM_001552.2 | -1.63 | Down | 0 |
| XM_017012260.1 | 1.65 | Up | 0 |
| NM_005345.5 | 1.50 | Up | 0 |
| XM_017010816.1 | 1.92 | Up | 0 |
| NM_001164097.1 | -1.70 | Down | 0 |
| NM_002508.2 | -1.02 | Down | 0 |
| NM_000760.3 | -1.13 | Down | 0 |
| XM_017001373.1 | -1.51 | Down | 0 |
| NM_005161.4 | -1.72 | Down | 0 |
| XM_011541872.2 | 4.33 | Up | 0 |
| NM_017709.3 | 2.27 | Up | 0 |
| NM_005505.4 | -1.15 | Down | 0 |
| XM_006714393.3 | -3.42 | Down | 0 |
| XM_011535014.1 | -1.64 | Down | 0 |
| NM_002117.5 | 3.69 | Up | 0 |
| NM_023068.3 | 3.96 | Up | 0 |
| NM_007118.3 | 1.27 | Up | 0 |
| NM_032777.9 | -1.16 | Down | 0 |
| NM_002781.3 | -4.41 | Down | 0 |
| NM_199187.1 | 1.17 | Up | 0 |
| XM_011514108.1 | -1.43 | Down | 0 |
| NM_024756.2 | -1.92 | Down | 0 |
| NM_001304815.1 | -3.33 | Down | 0 |
| NM_000735.3 | 1.67 | Up | 0 |
| XM_005264452.4 | -11.41 | Down | 0 |
| XM_005274245.1 | 1.50 | Up | 0 |
| NM_003242.5 | -2.09 | Down | 0 |
| NM_004684.5 | -1.38 | Down | 0 |
| NM_002253.2 | -1.15 | Down | 0 |
| NM_001005474.2 | 6.91 | Up | 0 |
| NM_020163.1 | -1.91 | Down | 0 |
| NM_030792.6 | -2.39 | Down | 0 |
| NM_005424.4 | -2.15 | Down | 0 |
| NM_001025109.1 | -1.13 | Down | 0 |
| XM_005250980.4 | -2.55 | Down | 0 |
| NM_001079877.2 | -2.79 | Down | 0 |
| XM_005258691.1 | 3.59 | Up | 0 |
| XM_011526660.2 | 3.01 | Up | 0 |
| XM_017026997.1 | -1.63 | Down | 0 |
| XM_005251972.3 | -3.65 | Down | 0 |
| NM_173157.2 | 5.72 | Up | 0 |
| XM_011544481.2 | 6.53 | Up | 0 |
| XM_011530710.2 | -1.32 | Down | 0 |
| NM_001297553.1 | 4.83 | Up | 0 |
| XM_005255182.3 | -1.57 | Down | 0 |
| XM_011541773.1 | 2.16 | Up | 0 |
| NM_003856.3 | -1.92 | Down | 0 |
| XM_006717523.2 | -2.34 | Down | 0 |
| NM_000130.4 | -1.78 | Down | 0 |
| XM_017001861.1 | -1.64 | Down | 0 |
| NM_005514.7 | 8.41 | Up | 0 |
| NM_002402.3 | -1.13 | Down | 0 |
| NM_001288705.1 | -1.08 | Down | 0 |
| XM_005255864.4 | -1.31 | Down | 0 |
| XM_011545721.2 | 3.35 | Up | 0 |
| NM_001014447.2 | -1.59 | Down | 0 |
| NM_152346.2 | -2.87 | Down | 0 |
| NM_057167.3 | -2.49 | Down | 0 |
| XM_006718057.3 | -2.33 | Down | 0 |
| XM_017010883.1 | -4.10 | Down | 0 |
| XM_006719363.1 | 4.90 | Up | 0 |
| NM_134269.2 | -3.09 | Down | 0 |
| NM_018243.3 | -1.76 | Down | 0 |
| XM_006710797.3 | -1.23 | Down | 0 |
| XM_005250983.2 | -2.12 | Down | 0 |
| XM_005257593.4 | 5.77 | Up | 0 |
| NM_001711.5 | -1.07 | Down | 0 |
| NM_017413.4 | -1.54 | Down | 0 |
| XM_017002421.1 | 1.91 | Up | 0 |
| NM_002444.2 | -1.05 | Down | 0 |
| NM_145753.2 | -1.35 | Down | 0 |
| NM_002507.3 | -1.89 | Down | 0 |
| XM_017004330.1 | -1.55 | Down | 0 |
| XM_017026994.1 | 1.75 | Up | 0 |
| NM_002153.2 | -1.04 | Down | 0 |
| NM_001773.2 | -1.61 | Down | 0 |
| XM_017001945.1 | -1.31 | Down | 0 |
| NM_001001391.1 | -2.77 | Down | 0 |
| NM_000032.4 | 2.25 | Up | 0 |
| NM_176819.3 | -1.33 | Down | 0 |
| XM_017005682.1 | -2.05 | Down | 0 |
| XM_011510202.1 | -5.14 | Down | 0 |
| NM_001322887.1 | -2.74 | Down | 0 |
| NM_001079823.1 | -1.17 | Down | 0 |
| NM_173800.4 | -1.25 | Down | 0 |
| XM_011523538.2 | 1.87 | Up | 0 |
| NM_020690.5 | -3.84 | Down | 0 |
| NM_004126.3 | -1.37 | Down | 0 |
| NM_001002274.2 | -1.19 | Down | 0 |
| NM_018406.6 | 6.95 | Up | 0 |
| NM_001077261.3 | -2.84 | Down | 0 |
| NM_001753.4 | -1.47 | Down | 0 |
| NM_001128848.1 | 2.71 | Up | 0 |
| NM_005116.5 | -1.42 | Down | 0 |
| NM_020991.3 | 7.02 | Up | 0 |
| XM_017010593.1 | -1.00 | Down | 0 |
| NM_014810.4 | -6.32 | Down | 0 |
| XM_005253704.4 | -4.26 | Down | 0 |
| NM_001198568.1 | -1.89 | Down | 0 |
| XM_017007902.1 | -2.99 | Down | 0 |
| NM_022369.3 | -2.05 | Down | 0 |
| NM_006904.6 | -1.10 | Down | 0 |
| NM_001542.3 | 3.45 | Up | 0 |
| XM_006713589.1 | 2.90 | Up | 0 |
| XM_011540222.2 | -2.69 | Down | 0 |
| XM_006716363.1 | -2.10 | Down | 0 |
| XM_005271081.3 | 1.10 | Up | 0 |
| NM_032242.3 | 1.06 | Up | 0 |
| NM_033043.1 | 1.97 | Up | 0 |
| NM_019055.5 | -1.07 | Down | 0 |
| NM_001242758.1 | -3.26 | Down | 0 |
| NM_001184772.2 | 2.06 | Up | 0 |
| NM_001289746.1 | -3.21 | Down | 0 |
| NM_030817.2 | -1.16 | Down | 0 |
| NM_001122659.2 | -2.19 | Down | 0 |
| XM_017017788.1 | 1.14 | Up | 0 |
| XM_011522528.2 | -2.08 | Down | 0 |
| NM_004184.3 | -1.22 | Down | 0 |
| NM_001128922.1 | -1.17 | Down | 0 |
| XM_017016115.1 | -1.03 | Down | 0 |
| XM_011532208.2 | 3.44 | Up | 0 |
| NM_032866.4 | -1.55 | Down | 0 |
| XM_005245764.1 | 3.75 | Up | 0 |
| XM_017002903.1 | 2.56 | Up | 0 |
| XM_005248151.3 | -6.48 | Down | 0 |
| XM_006724220.3 | 3.25 | Up | 0 |
| NM_001291411.1 | -2.24 | Down | 0 |
| NM_007183.3 | 2.18 | Up | 0 |
| XM_011527194.2 | 1.65 | Up | 0 |
| NM_004653.4 | 1.30 | Up | 0 |
| XM_005254034.3 | 2.57 | Up | 0 |
| NM_138440.2 | -1.14 | Down | 0 |
| NM_015852.3 | -1.66 | Down | 0 |
| XM_005250713.1 | -3.65 | Down | 0 |
| NM_000756.3 | 1.32 | Up | 0 |
| XM_005254248.2 | -1.97 | Down | 0 |
| NM_024832.4 | -4.10 | Down | 0 |
| NM_002780.4 | -1.57 | Down | 0 |
| NM_007173.5 | -1.65 | Down | 0 |
| XM_011528010.1 | 2.81 | Up | 0 |
| NM_001321788.1 | -5.18 | Down | 0 |
| XM_011511183.2 | 1.30 | Up | 0 |
| XM_017019839.1 | 4.77 | Up | 0 |
| NM_014918.4 | -1.15 | Down | 0 |
| NM_001193511.1 | 4.99 | Up | 0 |
| NM_001018011.1 | 5.48 | Up | 0 |
| NM_032801.4 | -1.15 | Down | 0 |
| XM_017016111.1 | 1.32 | Up | 0 |
| XM_005264187.3 | -4.45 | Down | 0 |
| XM_017012201.1 | 1.53 | Up | 0 |
| XM_017009238.1 | -1.32 | Down | 0 |
| NM_001080495.2 | -1.19 | Down | 0 |
| NM_005397.3 | -1.64 | Down | 0 |
| NM_007270.4 | -1.53 | Down | 0 |
| NM_033001.3 | -2.50 | Down | 0 |
| NM_001288576.1 | 2.43 | Up | 0 |
| NM_024574.3 | -1.37 | Down | 0 |
| XM_005251470.2 | 2.22 | Up | 0 |
| XM_011528247.1 | 3.05 | Up | 0 |
| NM_002410.4 | -2.20 | Down | 0 |
| NM_024940.7 | -1.66 | Down | 0 |
| NM_001166019.1 | -3.41 | Down | 0 |
| XM_017005272.1 | -2.19 | Down | 0 |
| XM_017019730.1 | 2.47 | Up | 0 |
| XM_011527061.2 | 4.97 | Up | 0 |
| XM_006721995.3 | -1.10 | Down | 0 |
| XM_011514452.2 | -3.00 | Down | 0 |
| NM_001039775.3 | -5.36 | Down | 0 |
| NM_006266.3 | -2.28 | Down | 0 |
| NM_174983.4 | -1.06 | Down | 0 |
| NM_198471.2 | -1.18 | Down | 0 |
| XM_011511422.1 | 3.16 | Up | 0 |
| XM_017027047.1 | -5.22 | Down | 0 |
| NM_000684.2 | -1.11 | Down | 0 |
| NM_001039703.5 | 1.07 | Up | 0 |
| XM_011509970.2 | -1.37 | Down | 0 |
| XM_017016710.1 | 3.56 | Up | 0 |
| NM_033238.2 | -2.69 | Down | 0 |
| NM_001004067.3 | -5.21 | Down | 0 |
| XM_005264787.2 | 2.18 | Up | 0 |
| NM_002116.7 | -2.04 | Down | 0 |
| NM_015491.2 | -2.87 | Down | 0 |
| NM_001161728.1 | -1.89 | Down | 0 |
| NM_001160125.1 | 2.13 | Up | 0 |
| NM_003773.4 | -1.23 | Down | 0 |
| XM_011538696.2 | 1.70 | Up | 0 |
| NM_000860.5 | -1.07 | Down | 0 |
| NM_006094.4 | 1.42 | Up | 0 |
| XM_011545030.1 | 5.95 | Up | 0 |
| NM_015150.1 | -1.83 | Down | 0 |
| NM_004689.3 | -2.09 | Down | 0 |
| XM_017005532.1 | 6.80 | Up | 0 |
| XM_017020516.1 | -1.58 | Down | 0 |
| NM_001113407.2 | -3.25 | Down | 0 |
| NM_021219.3 | -1.07 | Down | 0 |
| NM_000072.3 | -1.77 | Down | 0 |
| NM_001077206.3 | -1.08 | Down | 0 |
| XM_017028819.1 | 5.08 | Up | 0 |
| XM_017009212.1 | 1.79 | Up | 0 |
| NM_001003891.2 | -1.30 | Down | 0 |
| NM_001082959.1 | -1.23 | Down | 0 |
| XM_011533705.1 | 10.43 | Up | 0 |
| XM_011545060.2 | 2.59 | Up | 0 |
| XM_011513281.2 | -1.68 | Down | 0 |
| XM_005264071.2 | -8.52 | Down | 0 |
| NM_015397.3 | 1.19 | Up | 0 |
| XM_005251975.3 | -2.13 | Down | 0 |
| NM_053028.3 | -1.59 | Down | 0 |
| NM_003607.3 | -2.63 | Down | 0 |
| NM_001119.4 | -1.10 | Down | 0 |
| NM_004566.3 | -3.00 | Down | 0 |
| NM_001291862.2 | -1.60 | Down | 0 |
| XM_017000836.1 | -5.44 | Down | 0 |
| XM_017013733.1 | -2.98 | Down | 0 |
| NM_004592.3 | 2.15 | Up | 0 |
| NM_001083538.1 | 1.35 | Up | 0 |
| XM_017014266.1 | 1.16 | Up | 0 |
| XM_017024739.1 | -6.27 | Down | 0 |
| NM_001025930.3 | -3.15 | Down | 0 |
| XM_017029780.1 | -3.15 | Down | 0 |
| XM_006719529.3 | -1.20 | Down | 0 |
| XM_006719367.3 | -2.87 | Down | 0 |
| XM_006711451.3 | -2.19 | Down | 0 |
| XM_011517404.2 | -1.09 | Down | 0 |
| XM_017028861.1 | 4.41 | Up | 0 |
| XM_011538799.2 | -1.07 | Down | 0 |
| XM_005270794.4 | 3.12 | Up | 0 |
| XM_017007022.1 | -2.06 | Down | 0 |
| XM_017018215.1 | -1.87 | Down | 0 |
| XM_011514722.1 | -2.87 | Down | 0 |
| XM_017018598.1 | -1.54 | Down | 0 |
| XM_017022530.1 | -1.33 | Down | 0 |
| XM_005272620.3 | -3.91 | Down | 0 |
| NM_001184826.1 | 13.01 | Up | 0 |
| XM_017018257.1 | -3.56 | Down | 0 |
| NM_203416.3 | -1.49 | Down | 0 |
| NM_033631.3 | 1.75 | Up | 0 |
| XM_006720011.3 | 4.34 | Up | 0 |
| XM_011514042.2 | -6.19 | Down | 0 |
| NM_014220.2 | -1.39 | Down | 0 |
| NM_015401.4 | 10.89 | Up | 0 |
| XM_005256550.4 | 3.42 | Up | 0 |
| XM_005245718.2 | 1.34 | Up | 0 |
| NM_032450.2 | 2.11 | Up | 0 |
| NM_001321794.1 | 4.80 | Up | 0 |
| XM_011523768.2 | 1.44 | Up | 0 |
| NM_152421.3 | -3.00 | Down | 0 |
| XM_011542700.2 | -1.72 | Down | 0 |
| XM_017001897.1 | -1.27 | Down | 0 |
| NM_001684.4 | -1.12 | Down | 0 |
| NM_005794.3 | -1.94 | Down | 0 |
| NM_001286242.1 | 1.19 | Up | 0 |
| NM_001553.2 | -1.40 | Down | 0 |
| NM_006208.2 | -1.19 | Down | 0 |
| XM_011540345.1 | -4.23 | Down | 0 |
| NM_001144769.2 | 1.90 | Up | 0 |
| XM_011532852.2 | -1.67 | Down | 0 |
| NM_012193.3 | -1.16 | Down | 0 |
| NM_181489.5 | -2.37 | Down | 0 |
| NM_001256153.2 | 1.87 | Up | 0 |
| NM_001319051.1 | -1.46 | Down | 0 |
| XM_017012733.1 | 3.14 | Up | 0 |
| NM_006206.4 | -1.17 | Down | 0 |
| NM_015383.2 | 1.06 | Up | 0 |
| XM_005270680.2 | 3.52 | Up | 0 |
| NM_001199379.1 | -1.17 | Down | 0 |
| NM_015493.6 | -6.35 | Down | 0 |
| XM_005247002.3 | -6.54 | Down | 0 |
| NM_001157.2 | -2.37 | Down | 0 |
| XM_011510790.1 | -2.68 | Down | 0 |
| XM_011516934.2 | 5.58 | Up | 0 |
| NM_001159677.1 | -4.27 | Down | 0 |
| XM_011521839.2 | 1.82 | Up | 0 |
| NM_002587.4 | -1.34 | Down | 0 |
| XM_005259606.2 | -2.85 | Down | 0 |
| NM_024663.3 | -1.86 | Down | 0 |
| XM_005256463.2 | -1.32 | Down | 0 |
| NM_001261.3 | -1.36 | Down | 0 |
| XM_006716079.3 | -3.10 | Down | 0 |
| NM_001040092.2 | -1.06 | Down | 0 |
| NM_000906.3 | -1.16 | Down | 0 |
| NM_030926.5 | -1.01 | Down | 0 |
| XM_017006475.1 | 1.04 | Up | 0 |
| NM_020455.5 | -2.19 | Down | 0 |
| NM_030810.3 | -1.08 | Down | 0 |
| XM_005265427.3 | -1.77 | Down | 0 |
| NM_001113347.1 | -1.22 | Down | 0 |
| NM_001005619.1 | 2.29 | Up | 0 |
| XM_011536599.1 | -2.10 | Down | 0 |
| XM_006711112.2 | 3.47 | Up | 0 |
| NM_002074.4 | -1.82 | Down | 0 |
| NM_001142651.2 | -2.41 | Down | 0 |
| NM_057164.4 | -2.44 | Down | 0 |
| NM_030927.2 | -1.18 | Down | 0 |
| XM_017023112.1 | -4.51 | Down | 0 |
| NM_000618.4 | -1.87 | Down | 0 |
| NM_002571.3 | -8.83 | Down | 0 |
| XM_011511570.2 | -1.01 | Down | 0 |
| NM_001113239.2 | -1.08 | Down | 0 |
| NM_001025356.2 | -1.27 | Down | 0 |
| XM_005257412.3 | -1.12 | Down | 0 |
| NM_005245.3 | -1.24 | Down | 0 |
| NM_015088.2 | -1.52 | Down | 0 |
| XM_005268044.3 | -2.45 | Down | 0 |
| XM_017009689.1 | -5.79 | Down | 0 |
| NM_198392.2 | -1.57 | Down | 0 |
| NM_007056.2 | 1.27 | Up | 0 |
| XM_011529998.1 | -2.68 | Down | 0 |
| XM_017026540.1 | 5.89 | Up | 0 |
| NM_001199456.1 | 3.69 | Up | 0 |
| NM_182961.3 | -3.99 | Down | 0 |
| NM_003240.3 | -3.58 | Down | 0 |
| XM_005261995.3 | 3.98 | Up | 0 |
| XM_017027773.1 | 1.48 | Up | 0 |
| XM_011543155.2 | -3.34 | Down | 0 |
| NM_020962.2 | -5.09 | Down | 0 |
| XM_011538203.2 | -1.96 | Down | 0 |
| NM_002376.6 | -2.97 | Down | 0 |
| XM_017022005.1 | 4.68 | Up | 0 |
| NM_033183.2 | 1.68 | Up | 0 |
| NM_004863.3 | -1.17 | Down | 0 |
| NM_145294.4 | 1.97 | Up | 0 |
| XM_017004840.1 | 1.23 | Up | 0 |
| XM_017012837.1 | -12.83 | Down | 0 |
| NM_014730.3 | -1.06 | Down | 0 |
| NM_018003.2 | -2.86 | Down | 0 |
| NM_014654.3 | -2.20 | Down | 0 |
| XM_011541596.2 | 1.32 | Up | 0 |
| NM_001099789.1 | -1.76 | Down | 0 |
| NM_198320.3 | -1.14 | Down | 0 |
| NM_015568.3 | -2.36 | Down | 0 |
| XM_011518893.1 | -1.27 | Down | 0 |
| NM_007351.2 | -1.52 | Down | 0 |
| XM_005269235.2 | -2.77 | Down | 0 |
| NM_152673.3 | -1.83 | Down | 0 |
| XM_017007897.1 | -2.71 | Down | 0 |
| NM_001282101.1 | 2.03 | Up | 0 |
| XM_005273635.1 | -2.42 | Down | 0 |
| NM_001908.4 | -2.40 | Down | 0 |
| NM_018927.3 | -4.37 | Down | 0 |
| XM_017012152.1 | -1.25 | Down | 0 |
| XM_006717141.3 | -1.42 | Down | 0 |
| NM_021110.3 | -1.03 | Down | 0 |
| XM_017006393.1 | -2.47 | Down | 0 |
| XM_005262155.4 | -2.85 | Down | 0 |
| XM_011533262.1 | -6.00 | Down | 0 |
| XM_011523078.1 | 2.89 | Up | 0 |
| NM_001080517.2 | -1.80 | Down | 0 |
| NM_182776.2 | -3.18 | Down | 0 |
| NM_130464.2 | 1.14 | Up | 0 |
| XM_017018376.1 | 1.83 | Up | 0 |
| XM_017028362.1 | -2.71 | Down | 0 |
| NM_018920.3 | -3.28 | Down | 0 |
| XM_011545347.1 | 2.17 | Up | 0 |
| XM_005259152.4 | -1.50 | Down | 0 |
| NM_130476.2 | -2.07 | Down | 0 |
| NM_016434.3 | -2.72 | Down | 0 |
| XM_005259659.3 | -2.09 | Down | 0 |
| XM_017025269.1 | 1.36 | Up | 0 |
| XM_017018244.1 | -1.43 | Down | 0 |
| NM_001322468.1 | -1.41 | Down | 0 |
| XM_005246957.4 | 2.12 | Up | 0 |
| NM_032204.4 | 2.86 | Up | 0 |
| NM_001145796.1 | -2.96 | Down | 0 |
| NM_006107.3 | 1.15 | Up | 0 |
| NM_001193370.1 | -1.28 | Down | 0 |
| NM_052970.4 | -2.49 | Down | 0 |
| XM_017016708.1 | -1.96 | Down | 0 |
| XM_017011431.1 | 1.01 | Up | 0 |
| NM_198129.2 | -2.88 | Down | 0 |
| XM_006711052.3 | -1.42 | Down | 0 |
| XM_017008046.1 | 2.86 | Up | 0 |
| NM_001206747.1 | -2.16 | Down | 0 |
| NM_001282582.1 | -1.11 | Down | 0 |
| XM_011523482.1 | -4.40 | Down | 0 |
| XM_005274093.1 | -1.06 | Down | 0 |
| XM_017025904.1 | 12.53 | Up | 0 |
| XM_017005872.1 | -3.03 | Down | 0 |
| XM_005261696.1 | -1.94 | Down | 0 |
| NM_175060.2 | -1.54 | Down | 0 |
| NM_001642.2 | -2.42 | Down | 0 |
| XM_017029570.1 | -3.77 | Down | 0 |
| NM_004428.2 | -1.12 | Down | 0 |
| XM_017006017.1 | 2.67 | Up | 0 |
| XM_006721106.3 | 2.42 | Up | 0 |
| XM_005247172.2 | 12.50 | Up | 0 |
| NM_198834.2 | -3.30 | Down | 0 |
| XM_011543127.2 | -3.02 | Down | 0 |
| XM_006718698.1 | 3.29 | Up | 0 |
| XM_006715477.2 | -1.53 | Down | 0 |
| NM_012218.3 | 1.69 | Up | 0 |
| NM_001282858.1 | 2.22 | Up | 0 |
| XM_017003810.1 | 1.12 | Up | 0 |
| XM_017008010.1 | -2.42 | Down | 0 |
| XM_011534045.2 | 2.43 | Up | 0 |
| NM_001282180.1 | -4.76 | Down | 0 |
| XM_011511039.1 | 2.26 | Up | 0 |
| XM_006721994.3 | 1.28 | Up | 0 |
| NM_002246.2 | -1.30 | Down | 0 |
| XM_017006416.1 | -1.18 | Down | 0 |
| NM_002017.4 | -3.14 | Down | 0 |
| XM_017017388.1 | -1.94 | Down | 0 |
| NM_000757.5 | -1.16 | Down | 0 |
| XM_011537658.1 | 1.54 | Up | 0 |
| NM_009585.3 | -1.46 | Down | 0 |
| XM_011519609.2 | -3.64 | Down | 0 |
| NM_002135.4 | 6.78 | Up | 0 |
| NM_001145662.1 | -1.41 | Down | 0 |
| XM_011527804.2 | -1.82 | Down | 0 |
| NM_207171.2 | 2.63 | Up | 0 |
| XM_017016460.1 | -2.35 | Down | 0 |
| XM_017004556.1 | 2.50 | Up | 0 |
| NM_001323343.1 | 12.42 | Up | 0 |
| XM_017002342.1 | 5.51 | Up | 0 |
| XM_017020065.1 | 4.75 | Up | 0 |
| NM_032870.3 | 4.22 | Up | 0 |
| NM_201383.2 | -1.04 | Down | 0 |
| XM_011510549.2 | -2.11 | Down | 0 |
| NM_001199282.2 | 1.06 | Up | 0 |
| XM_011517203.1 | 1.18 | Up | 0 |
| XM_017019792.1 | 1.29 | Up | 0 |
| XM_005275331.2 | 1.62 | Up | 0 |
| XM_011530751.2 | 1.62 | Up | 0 |
| XM_017019788.1 | -3.15 | Down | 0 |
| XM_017000823.1 | -1.57 | Down | 0 |
| NM_012305.3 | -1.82 | Down | 0 |
| MTCONS_00063583 | 2.08 | Up | 0 |
| MTCONS_00154642 | 1.49 | Up | 0 |
| MTCONS_00126203 | 2.80 | Up | 0 |
| MTCONS_00126205 | 2.80 | Up | 0 |
| MTCONS_00126202 | 2.80 | Up | 0 |
| MTCONS_00126204 | 2.80 | Up | 0 |
| MTCONS_00063584 | 9.95 | Up | 0 |
| MTCONS_00126869 | -2.24 | Down | 0 |
| MTCONS_00075842 | 3.01 | Up | 0 |
| MTCONS_00114541 | 1.46 | Up | 0 |
| MTCONS_00053712 | -1.31 | Down | 0 |
| MTCONS_00322406 | -4.12 | Down | 0 |
| MTCONS_00321306 | -2.32 | Down | 0 |
| MTCONS_00178613 | 1.66 | Up | 0 |
| MTCONS_00000060 | -1.34 | Down | 0 |
| MTCONS_00067892 | 3.78 | Up | 0 |
| MTCONS_00299886 | -1.19 | Down | 0 |
| MTCONS_00118885 | 1.14 | Up | 0 |
| MTCONS_00178546 | 1.78 | Up | 0 |
| MTCONS_00295667 | -3.22 | Down | 0 |
| MTCONS_00322533 | -2.31 | Down | 0 |
| MTCONS_00123612 | -1.97 | Down | 0 |
| MTCONS_00144115 | -1.20 | Down | 0 |
| MTCONS_00340850 | 1.29 | Up | 0 |
| MTCONS_00106256 | 1.07 | Up | 0 |
| MTCONS_00144108 | -1.54 | Down | 0 |
| MTCONS_00095756 | 2.27 | Up | 0 |
| MTCONS_00022491 | -1.31 | Down | 0 |
| MTCONS_00015690 | -2.33 | Down | 0 |
| MTCONS_00178600 | 2.43 | Up | 0 |
| MTCONS_00019163 | -1.57 | Down | 0 |
| MTCONS_00352172 | 1.63 | Up | 0 |
| MTCONS_00226508 | 1.32 | Up | 0 |
| MTCONS_00178620 | -1.23 | Down | 0 |
| MTCONS_00365501 | -1.28 | Down | 0 |
| MTCONS_00005577 | -1.11 | Down | 0 |
| MTCONS_00256755 | -1.65 | Down | 0 |
| MTCONS_00317494 | -1.42 | Down | 0 |
| MTCONS_00309408 | 3.73 | Up | 0 |
| MTCONS_00085589 | 2.19 | Up | 0 |
| MTCONS_00133176 | -1.31 | Down | 0 |
| MTCONS_00381358 | 4.41 | Up | 0 |
| MTCONS_00133181 | -1.70 | Down | 0 |
| MTCONS_00086341 | -1.62 | Down | 0 |
| MTCONS_00150453 | 3.35 | Up | 0 |
| MTCONS_00341797 | -8.78 | Down | 0 |
| MTCONS_00318663 | -5.41 | Down | 0 |
| MTCONS_00034169 | -4.27 | Down | 0 |
| MTCONS_00030352 | 3.96 | Up | 0 |
| MTCONS_00122488 | -2.69 | Down | 0 |
| MTCONS_00107045 | 5.02 | Up | 0 |

FC, fold change.

Table S3: The 160 significant proteins between placenta accreta and control group in proteome.

| Protein ID | log_2_FC | Regulation Trend | *P* value |
| --- | --- | --- | --- |
| F118A | -1.58 | Down | 0 |
| ALKB4 | 0.62 | Up | 0 |
| MECP2 | -3.77 | Down | 0.001 |
| QSPP | 0.83 | Up | 0.001 |
| KCMF1 | 1.23 | Up | 0.001 |
| TBB8 | -0.65 | Down | 0.003 |
| POGZ | -1.66 | Down | 0.003 |
| TXN4A | -1.5 | Down | 0.005 |
| CASC3 | 0.99 | Up | 0.005 |
| GLNA | -0.93 | Down | 0.006 |
| DYL1 | -0.81 | Down | 0.006 |
| SKIV2 | -0.65 | Down | 0.006 |
| LSM7 | -1.15 | Down | 0.006 |
| MTMR5 | 2.62 | Up | 0.006 |
| IMPCT | 0.63 | Up | 0.006 |
| C1S | -0.61 | Down | 0.007 |
| NXN | -1.3 | Down | 0.007 |
| IFG15 | 0.76 | Up | 0.007 |
| FBRL | -1.02 | Down | 0.008 |
| TRM6 | -0.89 | Down | 0.008 |
| HERC1 | 0.91 | Up | 0.008 |
| H90B4 | -0.65 | Down | 0.01 |
| HYCCI | 1.27 | Up | 0.01 |
| 5NTD | -0.62 | Down | 0.011 |
| INHBC | -2.48 | Down | 0.011 |
| IMA5 | -1.3 | Down | 0.012 |
| CON__P13646-1 | 1.48 | Up | 0.012 |
| CSF1R | 0.81 | Up | 0.012 |
| UBP11 | -0.9 | Down | 0.013 |
| ZFY16 | -0.92 | Down | 0.013 |
| PROZ | 2.3 | Up | 0.013 |
| CTGE5 | -1.3 | Down | 0.014 |
| RL9 | -0.73 | Down | 0.014 |
| RL22 | -0.73 | Down | 0.014 |
| MTREX | -0.87 | Down | 0.014 |
| COMD3 | -1.1 | Down | 0.014 |
| IRF6 | 0.87 | Up | 0.014 |
| BCCIP | 0.96 | Up | 0.014 |
| PDIP3 | -1.76 | Down | 0.015 |
| IPO13 | -0.96 | Down | 0.016 |
| RL32 | -0.96 | Down | 0.017 |
| KPCD | -1.42 | Down | 0.017 |
| CBR1 | 1.15 | Up | 0.017 |
| YAP1 | 0.81 | Up | 0.017 |
| PABP4 | -0.86 | Down | 0.018 |
| EGFLA | -0.83 | Down | 0.018 |
| UMPS | 1.01 | Up | 0.018 |
| HIP1 | -0.78 | Down | 0.019 |
| LAMA5 | -0.69 | Down | 0.019 |
| CLIC2 | -1.5 | Down | 0.019 |
| RS5 | -0.61 | Down | 0.019 |
| DHRS4 | -2.34 | Down | 0.019 |
| KCRU | -1.27 | Down | 0.02 |
| TFAM | -1.08 | Down | 0.02 |
| TPBG | -0.71 | Down | 0.02 |
| CHMP7 | 1.12 | Up | 0.02 |
| LPAR1 | 2.27 | Up | 0.02 |
| TEN3 | -1.26 | Down | 0.021 |
| SNX29 | 1.42 | Up | 0.021 |
| PLXB2 | -0.99 | Down | 0.022 |
| FRG1 | -1.08 | Down | 0.022 |
| BRAT1 | -1.28 | Down | 0.022 |
| KCC2A | -0.66 | Down | 0.022 |
| CYH3 | -0.74 | Down | 0.023 |
| MFGM | -0.68 | Down | 0.023 |
| RL27 | -1.39 | Down | 0.024 |
| TRIA1 | 0.75 | Up | 0.024 |
| ARGI1 | 1.01 | Up | 0.024 |
| NEBL | -0.89 | Down | 0.025 |
| FLNC | -1 | Down | 0.025 |
| CBPZ | -1.3 | Down | 0.025 |
| DLG3 | -0.84 | Down | 0.025 |
| RL36 | -1.21 | Down | 0.026 |
| FBP1L | 0.69 | Up | 0.026 |
| RL17 | -1.25 | Down | 0.027 |
| MTA3 | -1.14 | Down | 0.027 |
| ERGI3 | -0.6 | Down | 0.027 |
| CRF | 2.16 | Up | 0.027 |
| EGLN | -0.6 | Down | 0.028 |
| NECT4 | 0.72 | Up | 0.028 |
| MYO1C | -0.63 | Down | 0.029 |
| RL14 | -0.85 | Down | 0.029 |
| H33 | -2.9 | Down | 0.029 |
| GET4 | -0.9 | Down | 0.029 |
| AN32A | -0.88 | Down | 0.03 |
| SVEP1 | -0.67 | Down | 0.03 |
| GOGA7 | -0.78 | Down | 0.03 |
| CP11A | -0.85 | Down | 0.031 |
| RS14 | -0.84 | Down | 0.031 |
| S27A4 | -0.79 | Down | 0.031 |
| SMRD2 | -1.31 | Down | 0.031 |
| TAP2 | 1.59 | Up | 0.031 |
| NALD2 | 0.9 | Up | 0.031 |
| APOD | -0.82 | Down | 0.032 |
| RL5 | -0.7 | Down | 0.032 |
| MYG1 | 0.68 | Up | 0.032 |
| PODN | -0.81 | Down | 0.033 |
| CC127 | -1.13 | Down | 0.033 |
| SYYM | -0.77 | Down | 0.033 |
| NT5D1 | 0.72 | Up | 0.033 |
| FKBP5 | -0.79 | Down | 0.035 |
| ARMC8 | -0.75 | Down | 0.035 |
| MGT5A | 0.76 | Up | 0.035 |
| NISCH | 0.65 | Up | 0.035 |
| H2B1J | -0.98 | Down | 0.036 |
| CETP | -0.64 | Down | 0.036 |
| TM201 | -1.3 | Down | 0.036 |
| MIC27 | -0.88 | Down | 0.036 |
| ASAP1 | 0.86 | Up | 0.036 |
| RL4 | -1.06 | Down | 0.037 |
| CD81 | -0.7 | Down | 0.037 |
| RL37A | -0.91 | Down | 0.037 |
| MICU3 | -1.04 | Down | 0.037 |
| SEC13 | -0.59 | Down | 0.038 |
| RS30 | -1.32 | Down | 0.038 |
| DCTN5 | -1.01 | Down | 0.038 |
| RL7A | -1.1 | Down | 0.039 |
| IQGA2 | -0.64 | Down | 0.039 |
| FA98B | -0.78 | Down | 0.039 |
| AP4B1 | -0.79 | Down | 0.039 |
| PIN4 | 0.63 | Up | 0.039 |
| PHIPL | -0.88 | Down | 0.04 |
| GAR1 | -0.76 | Down | 0.04 |
| CLCB | -0.74 | Down | 0.041 |
| SRP14 | -0.67 | Down | 0.041 |
| CDAN1 | 0.85 | Up | 0.041 |
| OPHN1 | -0.66 | Down | 0.042 |
| RL8 | -1.45 | Down | 0.042 |
| RL18 | -1.05 | Down | 0.042 |
| H1X | -1.46 | Down | 0.042 |
| EHD2 | -0.74 | Down | 0.042 |
| TIM8A | -0.63 | Down | 0.043 |
| PA24A | -0.81 | Down | 0.043 |
| PAXI | -0.75 | Down | 0.043 |
| RL3 | -0.85 | Down | 0.044 |
| ZGRF1 | -0.64 | Down | 0.044 |
| RL27A | -0.93 | Down | 0.045 |
| RS4X | -0.82 | Down | 0.045 |
| FHL3 | -0.84 | Down | 0.045 |
| PRP19 | -0.86 | Down | 0.045 |
| LAS1L | -1.15 | Down | 0.045 |
| JUND | -0.78 | Down | 0.046 |
| APC15 | -1.64 | Down | 0.046 |
| RL38 | -1.02 | Down | 0.046 |
| PSG5 | -0.59 | Down | 0.046 |
| NUP93 | -0.8 | Down | 0.046 |
| SMHD1 | 0.68 | Up | 0.046 |
| LDHA | 0.59 | Up | 0.046 |
| RPAB1 | -0.86 | Down | 0.047 |
| ACTG | -1.14 | Down | 0.047 |
| DHB8 | -0.9 | Down | 0.047 |
| GRHL2 | -1.93 | Down | 0.048 |
| LIMC1 | -1.17 | Down | 0.048 |
| CBR3 | 0.97 | Up | 0.048 |
| K2C5 | 0.82 | Up | 0.048 |
| ARF6 | -0.59 | Down | 0.049 |
| GLRX5 | -0.68 | Down | 0.049 |
| HS12B | -0.9 | Down | 0.049 |
| PTPRJ | -0.69 | Down | 0.05 |
| SPCS1 | -0.63 | Down | 0.05 |

FC, fold change.

Table S4. The gestational week at which samples were collected.

| Samples | Gestational weeks |
| --- | --- |
| Control 1 | 36.57 |
| Control 2 | 36.57 |
| Control 3 | 37.14 |
| Control 4 | 36.29 |
| Control 5 | 36.00 |
| Placenta accreta 1 | 36.00 |
| Placenta accreta 2 | 37.14 |
| Placenta accreta 3 | 36.00 |
| Placenta accreta 4 | 37.14 |
| Placenta accreta 5 | 36.43 |
